# Bone metastases in patients with metastatic renal cell carcinoma: are they always associated with poor prognosis?

**DOI:** 10.1186/s13046-015-0122-0

**Published:** 2015-02-05

**Authors:** Matteo Santoni, Alessandro Conti, Giuseppe Procopio, Camillo Porta, Toni Ibrahim, Sandro Barni, Francesco Maria Guida, Andrea Fontana, Alfredo Berruti, Rossana Berardi, Francesco Massari, Bruno Vincenzi, Cinzia Ortega, Davide Ottaviani, Giacomo Carteni, Gaetano Lanzetta, Delia De Lisi, Nicola Silvestris, Maria Antonietta Satolli, Elena Collovà, Antonio Russo, Giuseppe Badalamenti, Stefano Luzi Fedeli, Francesca Maria Tanca, Vincenzo Adamo, Evaristo Maiello, Roberto Sabbatini, Alessandra Felici, Saverio Cinieri, Rodolfo Montironi, Sergio Bracarda, Giuseppe Tonini, Stefano Cascinu, Daniele Santini

**Affiliations:** Department of Medical Oncology, AOU Ospedali Riuniti, Università Politecnica delle, Marche, Ancona Italy; Department of Clinical and Specialist Sciences, Urology, Università Politecnica delle Marche, Ancona, Italy; Department of Medical Oncology, Fondazione IRCCS Istituto Nazionale dei Tumori, Milan, Italy; Division of Medical Oncology, I.R.C.C.S. San Matteo University Hospital Foundation, Pavia, Italy; Osteoncology and Rare Tumors Center, IRCCS Istituto Scientifico Romagnolo per lo Studio e la Cura dei Tumori (IRST), Meldola, FC Italy; Medical Oncology Department, Azienda Ospedaliera Treviglio-Caravaggio, Treviglio, Italy; Department of Medical Oncology, Campus Bio-Medico University of Rome, Rome, Italy; Unit of Medical Oncology 2, Istituto Toscano Tumori, Azienda-Ospedaliero-Universitaria Pisana, Pisa, Italy; Dipartimento di Specialità Medico-Chirurgiche, Medical Oncology, Scienze Radiologiche e Sanità Pubblica, Università degli Studi di Brescia, Azienda Ospedaliera Spedali Civili, Brescia, Italy; Department of Medical Oncology, “G.B. Rossi” Academic Hospital, Azienda Ospedaliera Universitaria Integrata, University of Verona, Verona, Italy; Department of Medical Oncology, Institute for Cancer Research & Treatment (IRCC), Candiolo, Torino Italy; Department of Medical Oncology, Presidio Sanitario Gradenigo, Turin, Italy; Department of Medical Oncology, Cardarelli Hospital, Naples, Italy; Department of Neurological Sciences, Neuromed Institute, IRCSS, Pozzilli, IS Italy; Istituto Neurotraumatologico Italiano, Unità Funzionale di Oncologia, Grottaferrata, Italy; Medical Oncology Unit, National Cancer Research Centre “Giovanni Paolo II”, Bari, Italy; Department of Oncology, University of Turin, Medical Oncology 1, AOU Città della Salute e della Scienza, Turin, Italy; Division of Medical Oncology, Hospital of Legnano, Milan, Italy; Department of Surgery and Oncology, Section of Medical Oncology, University of Palermo, Palermo, Italy; Department of Medical Oncology, AOU Ospedali Riuniti, Università Politecnica delle Marche, Presidio San Salvatore, Pesaro, Italy; Department of Medical Oncology, University of Cagliari, Cagliari, Italy; Department of Human Pathology, Medical Oncology Unit AOOR Papardo-Piemonte, University of Messina, Messina, Italy; Oncology Unit, IRCCS Casa Sollievo della Sofferenza, San Giovanni Rotondo, FG Italy; Dipartimento Integrato di Oncologia ed Ematologia, Medical Oncology Division, Università degli Studi di Modena e Reggio Emilia, Modena, Italy; Department of Medical Oncology, Regina Elena National Cancer Institute, Rome, Italy; Medical Oncology Department & Breast Unit - Hospital of Brindisi and Medical Oncology Department - European Institute of Oncology, Milan, Italy; Section of Pathological Anatomy, Polytechnic University of the Marche Region, School of Medicine, United Hospitals, Ancona, Italy; Department of Oncology, USL-8, Ospedale San Donato, Arezzo, Italy

**Keywords:** Bone metastasis, Prognostic factors, Renal cell carcinoma, Time to distant metastasis

## Abstract

**Purpose:**

Aim of this study was to investigate for the presence of existing prognostic factors in patients with bone metastases (BMs) from RCC since bone represents an unfavorable site of metastasis for renal cell carcinoma (mRCC).

**Materials and methods:**

Data of patients with BMs from RCC were retrospectively collected. Age, sex, ECOG-Performance Status (PS), MSKCC group, tumor histology, presence of concomitant metastases to other sites, time from nephrectomy to bone metastases (TTBM, classified into three groups: <1 year, between 1 and 5 years and >5 years) and time from BMs to skeletal-related event (SRE) were included in the Cox analysis to investigate their prognostic relevance.

**Results:**

470 patients were enrolled in this analysis. In 19 patients (4%),bone was the only metastatic site; 277 patients had concomitant metastases in other sites. Median time to BMs was 16 months (range 0 − 44y) with Median OS of 17 months. Number of metastatic sites (including bone, *p* = 0.01), concomitant metastases, high Fuhrman grade *(p < 0.001)* and non-clear cell histology (*p* = 0.013) were significantly associated with poor prognosis. Patients with TTBM >5 years had longer OS (22 months) compared to patients with TTBM <1 year (13 months) or between 1 and 5 years (19 months) from nephrectomy *(p < 0.001),* no difference was found between these two last groups *(p = 0.18*). At multivariate analysis, ECOG-PS, MSKCC group and concomitant lung or lymph node metastases were independent predictors of OS in patients with BMs.

**Conclusions:**

Our study suggest that age, ECOG-PS, histology, MSKCC score, TTBM and the presence of concomitant metastases should be considered in order to optimize the management of RCC patients with BMs.

## Introduction

Renal cell carcinoma (RCC) accounts for approximately 5% of epithelial cancers worldwide with clear cell RCC representing 85% of these tumors [1]. Almost one third of patients present with synchronous metastatic disease and another 20% experience recurrence or develop metastatic RCC (mRCC) after nephrectomy [2,3].

The introduction of tyrosine kinase (TKIs) and mTOR inhibitors has completely revolutionized the therapeutic scenario of mRCC, suddenly replacing immunotherapy as the standard of care for these patients. Seven agents have been approved by the US Food and Drug Administration (FDA), starting from sorafenib in 2005, followed by sunitinib, bevacizumab plus interferon, everolimus, temsirolimus, pazopanib and axitinib. However, new clinical and molecular predictive and prognostic biomarkers are dramatically required in order to optimize the use of novel effective agents for mRCC.

Bone metastases (BMs) occurs in almost 35% of patients with advanced RCC [4]. Although the management of patients with BMs has been markedly improved by the introduction of bone-directed targeted therapies, their prognosis is still dismal, with a mean survival of 12 months [4-6]. Skeletal involvement is commonly an aggressive, lytic process which causes substantial morbidity through skeletal related events (SREs), defined as a pathological fracture, surgical intervention, requirement for palliative radiotherapy to bone, spinal cord compression or hypercalcemia. Almost 70% of RCC patients with BMs experience at least one SRE [7].

**Figure 1:**
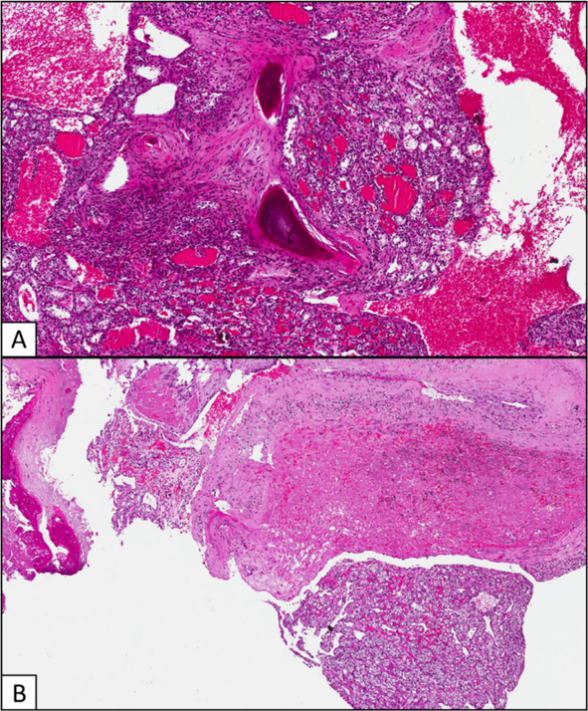
**Bone metastases occurred at time of clear cell RCC diagnosis (A) and 12 years after radical nephrectomy (B).**

The probability to develop BMs in RCC patients parallels with increased survival related to the introduction of biological therapies (Figure 1). Several studies suggest that the presence of BMs is associated with poor prognosis [8,9]. BMs are usually related to a more aggressive subtype of disease as suggested by the higher percentage of patients with metastases or Fuhrman grade 4 at the initial diagnosis, the shorter median time between nephrectomy and diagnosis of metastatic disease and the greater number of metastatic sites at the diagnosis [10]. However, long survival in patients with BMs from RCC is not a rare event. This may be partially explained by data on tumor biological heterogeneity [11,12], although other factors may affect the natural history of BMs in RCC population. Aim of this study was to investigate for the presence of existing prognostic factors in a large cohort of patients with BMs from RCC.

### Patients and methods

Data were extracted from the databases of 19 Italian centers involved in the treatment of patients with mRCC. Inclusion criteria were: histologic diagnosis of RCC, clinical diagnosis of BMs, regularly conducted follow-up of the disease, minimum uneventful follow-up of 1 year after diagnosis of metastasis or alternatively known death due to the disease. Pts were excluded from the analysis if they had missing information about sites of metastasis or time to bone metastasis (TTBM).

Patients characteristics and clinic-pathological variables considered in this study were: gender, age, eastern cooperative oncology group performance status (ECOG-PS), tumor histology, clinical stage at time of surgery, time from surgery to diagnosis of BMs, MSKCC risk-group, type and number of sites of metastasis, time from BM to development of further metastatic sites and presence/type of SREs. Since we gathered data from RCC patients treated from 2001 onwards, we were not able to categorize all of them according to the novel IMDC (or Heng’s) prognostic classification.

BMs were assessed with bone scan and confirmed by contrast-enhanced CT, while other metastatic sites were defined either with total body contrast-enhanced CT or MRI.

Cancer-specific survival was computed both from any site-metastasis and from BM to the event (death) and both measurements were considered as outcome measures.

Continuous covariates (age, TTBM) were grouped into discrete ordinal categories. Age was divided in two ordinal groups (<65 years and ≥65 years). Correlation between continuous variables was assessed by means of Pearson product-limit correlation coefficient [12]. As outcome variables, the overall survival (OS) from the diagnosis of BMs was analyzed.

Patients were grouped according to TTBM: Group A (<1 year), Group B (between 1 and 5 years) and Group C (>5 years). The choice of these cut-offs was related to the reported data on the prognostic effects of early (within 1 year) or late (>5 years) time to metastasis on the outcome of RCC patients [13-16].

Survival analysis was conducted via the Kaplan-Meier method and Mantel-Haenszel log-rank test was employed to compare survival among groups. A Cox-regression model was applied to the data with a univariate and multivariate approach. The assumption of proportionality of hazards was assessed using the Therneau and Grambsch test of the Schönefeld residuals [17]. Variables not fitting at univariate regression analysis were excluded for the multivariate model. No-multicollinearity of the grouped co-variates was also checked. Significance level in the univariate model for inclusion in the multivariate final model was more liberally set at a 0.2 level, according to Hosmer *et al.* [17]. All other significance levels were set at a 0.05 value. Statistical analysis was conducted with R –Software version 3.0.1 (The R Company – Vienna –Austria).

## Results

We retrospectively collected clinical data of 511 patients with RCC BMs from 19 Italian Institutions followed between January 2001 and April 2014. Of 470 patients were available for this analysis (41 were excluded due to lack of data on BMs or follow-up). Median age was 65 years (range 30 to 92 years). Three hundred and thirty-five patients (71%) were males; 398 (85%) had clear cell RCC, whereas 72 (15%) presented with other histologies (5% papillary, 1% chromophobe, 9% other). The complete list of patient characteristics is shown in Table 1.

**Table 1:** **Patient demographics and disease characteristics**

	**Patients, n (%) (** ***N*** **= 470)**
**Median age, y (range)**	65 (30–92)
**Sex**	
Male	335 (71)
Female	135 (29)
**Tumor histology**	
Clear cell	398 (85)
Papillary	25 (5)
Chromophobe	5 (1)
Other	42 (9)
**ECOG-Performance Status (PS) ≥2**	60 (13)
**Time to distant metastases (TTBM)**	
Group A (<1 year)	229 (49)
Group B (between 1 and 5 years)	107 (23)
Group C (>5 years)	134 (28)
**MSKCC criteria**	
Good	198 (42)
Intermediate	219 (47)
Poor	53 (11)
**Median number of bone metastases**	2
**Median number of SREs (range)**	2 (1–6)
**Sites of concomitant metastases**	
Lung	276 (59)
Lymph node	205 (44)
Liver	78 (17)
Brain	30 (6)
Adrenal gland	14 (3)
Treatment after onset of bone metastases	190 (41)
Sunitinib	61(12)
Sorefnib	4 (1)
Pazopanib	24 (5)
mTor inhibitors	191 (41)
Other treatments or no treatemnt	

Median number of bone metastases was 2. Median number of SREs was 1 (range 0 to 6 events). The median TTBM was 16 months (95% CI 0 – 64). Median time to first SRE was 2 months (95% CI 0 – 4) from BM diagnosis. Median OS was 17 months (95% CI 14 to 19).

Prognostic categories using MSKCC criteria were good in 198 pts (42%), intermediate in 219 (47%) and poor in 53 (11%). The median OS was 22 (95% CI 20 to 32), 18 (95% CI 14 to 22) and 7 months (95% CI 6 to 10) in patients with good, intermediate and poor prognosis, respectively *(p < 0.001).*

**Figure 2:**
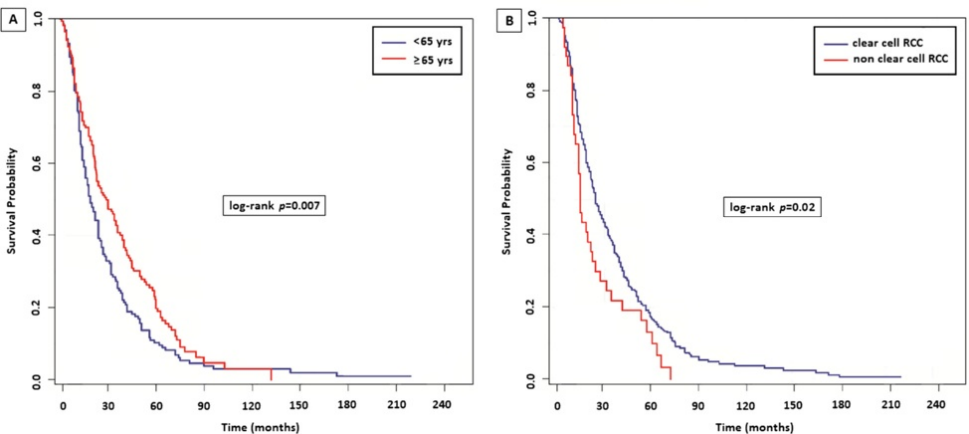
**Overall Survival (OS) from the diagnosis of bone metastases (BMs) based on age (2A) and histology (2B).**

A significantly higher mortality both from the diagnosis of mRCC and BMs was observed in patients developing metastases < 65y compared to ≥ 65 (median OS: 15 months (95% CI 13 to 20) vs 21 months (95% CI 18 to 24), *p* = 0.038 (Figure 2A). No differences in OS were observed based on sex *(p = 0.31).*

Clear-cell histology was correlated with a longer survival as compared with other histologic types (18 months [95% CI 15 to 20] vs 12 months [95% CI 8 to 18], *p* = 0.014 Figure 2B), but this observation is someway hampered by the small number of patients with non-clear cell histologies and the heterogeneous natural history of the different histotypes. Also ECOG-PS at time of diagnosis of metastatic disease was related to patients’ survival at univariate analysis (log-rank *p < 0.001*; HR: 1.22, 95% CI 1.05-1.40*, p = 0.007).*

The number of BMs was associated neither with the number of SREs *(p = 0.096)* nor with OS (HR: 1.17, 95% CI 0.92-1.43, *p = 0.21*). No significant differences in terms of OS were found when comparing patients presenting with visceral metastases as first metastatic sites with those with BMs as first metastatic site (HR: 1.18, 95% CI 0.89 to 1.54*, p = 0.25*).

**Figure 3:**
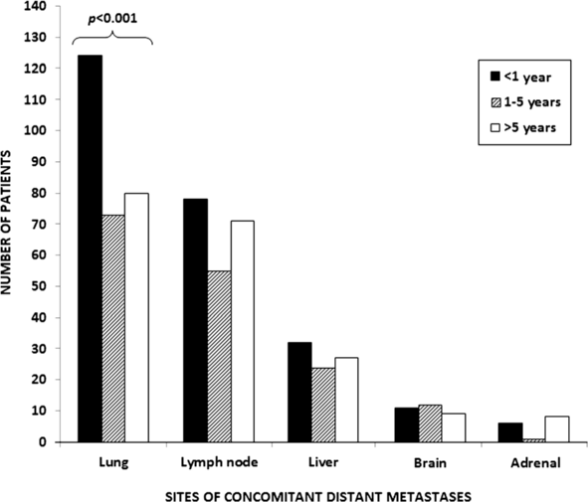
**Distribution of sites of concomitant distant metastases in patients with bone metastases (BMs).**

Furthermore, we analyzed the prognostic role of concomitant visceral metastases in patients with BMs. Two-hundred seventy-six patients (59%) had concomitant lung metastases, 205 (44%, with 28% retroperitoneal, 12% mediastinal and 4% to other sites) had metastases to lymph nodes and 78 (17%) had liver metastases (Figure 3). Interestingly, the number and site of concomitant metastases was significantly associated with OS *(p = 0.016)* in our population. The presence of concomitant lung *(p = 0.012),* liver *(p = 0.005),* or lymph-node *(p = 0.014)* metastases was significantly correlated with OS at univariate analysis, while no correlation was shown for brain *(p = 0.65)* or adrenal metastases *(p = 0.374)* (Table 2).

**Table 2:** **Univariate and multivariable analysis of predictors of OS from the diagnosis of bone metastases (BMs) in patients with RCC**

	***UVA*** *			***MVA*** **		
	***HR***	***95% CI***	**P**	***HR***	***95% CI***	***P***
Age (<65 vs ≥65)	0.8	0.64-0.99	0.04	0.83	0.65-1.07	*0.15*
Gender (M vs F)	0.84	0.68-1.04	0.31			
Histology (CC vs NCC)	1.45	1.08-1.95	0.013	1.23	0.83-1.81	*0.31*
MSKCC group	1.94	1.64-2.29	<0.001	1.82	1.50-2.20	***<0.001***
ECOG-PS	1.44	1.26-1.64	<0.001	1.40	1.19-1.66	***<0.001***
Time from diagnosis of primary disease (<1 y - 1-5y - >5 y)	0.72	0.64-0.81	<0.001	1.00	0.83-1.21	*0.97*
Number of bone metastases (single- vs multiple sites)	1.15	0.92-1.43	0.21			
Number of other sites of metastasis	1.17	1.04-1.32	0.011	1.06	0.78-1.44	*0.70*
**Other sites of metastasis**						
Local recurrence	1.05	0.76-1.46	0.78			
Lung	1.19	0.96-1.48	0.012	1.41	1.09-1.84	***0.009***
Liver	1.43	1.11-1.84	0.005	1.2	0.87-1.66	*0.26*
Lymph-nodes	1.30	1.06-1.59	0.014	1.30	1.02-1.67	***0.03***
Adrenals	0.77	0.43-1.37	0.374			
Brain	0.91	0.62-1.35	0.65			
Other sites	0.60	0.37-0.97	0.04	0.59	0.32-1.09	*0.09*

HR and significance levels of significant variables are given as computed after removal of non-significant covariates. *UVA: Univariate analysis. **MVA: Multivariate analysis.

cc = clear cell; ECOG-PS = Eastern Cooperative Oncology Group Performance Status; F = female; bold p value represents independent prognostic factor for OS.

M = male; MSKCC = Memorial Sloan Kettering Cancer Center.

At multivariate analysis, MSKCC risk group (*p < 0.001*), ECOG-PS *(p < 0.001),* lymph-node *(p = 0.03)* and lung *(p = 0.009)* metastases were independent prognostic factors for OS (Table 2).

Patients were grouped according to TTBM: Group A (<1 year, 229 patients), Group B (between 1 and 5 years, 107 patients) and Group C (>5 years, 134 patients). The number of metastatic sites was statistically different in the three groups, with a median of 1 site in Group A, 2 In Group B and 2 in Group C *(p = 0.001*). TTBM was inversely correlated with MSKCC risk group (testing for difference between groups with non-homogeneous variances, Mann–Whitney U = 97, *p < 0.001*, Kendal tau = −0.40, *p < 0.001*). Indeed, patients with longer TTBM presented more frequently good risk features.

A significant difference was found for the distribution of lung metastases (*p < 0.001*, Figure 3), with a higher incidence in Group B compared to the other groups. No significant differences were found for the incidence of lymph-node *(p = 0.20),* liver *(p = 0.24),* adrenal, brain metastases *(p = 0.15)* or local recurrence *(p = 0.75).*

**Figure 4:**
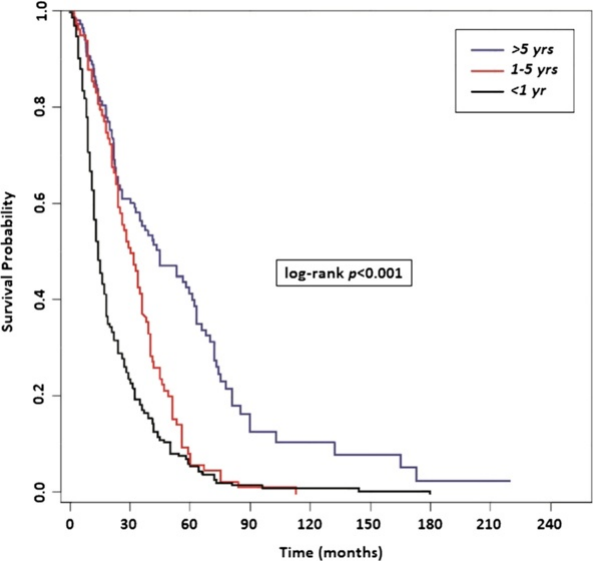
**Overall survival (OS) from the diagnosis of bone metastases (BMs) based on the time of bone recurrence from nephrectomy (TTBM).**

As regard to OS, no significant difference was found between Group A and B (13 months [95% CI 12 to 15] vs 19 months [95% CI 12 to 26], *p* = 0.36), while significant differences were found when comparing Group A with Group C (13 months [95% CI 12 to 15] vs 22 months [95% CI 20 to 33] *p < 0.001)* and Group B with Group C (19 months [95% CI 12 to 26] vs 22 months [95% CI 20 to 33] *p < 0.001)* (Figure 4).

**Figure 5:**
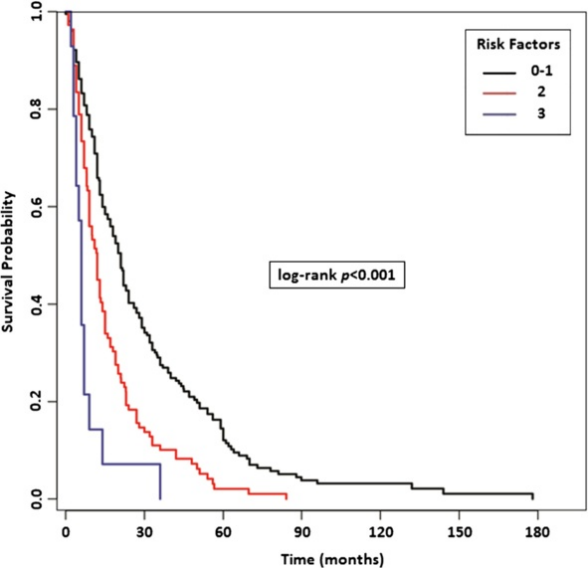
**Risk Stratification Model in patients with bone metastases (BMs) based on the presence of significant prognostic factors resulted from multivariate analysis (MSKCC risk, ECOG-PS, lymph-node and/or lung metastases).**

Finally, we identified three risk categories: the favorable risk group (Risk Score = 0–1 of previous confirmed three prognostic factors, median OS = 21 months), the intermediate risk group (Risk Score = 2, median OS = 12 months) and the poor risk group (Risk Score = 3, median OS = 6 months) (*p < 0.001*, HR 1.91, 95% CI: 1.56-2.34, Figure 5).

## Discussion

BMs are common in patients with mRCC, but the mechanism by which this tumor preferentially metastasize to bone is poorly understood. The development of BMs is a multifactorial process that requires a series of interactions between invading tumor cells and the bone microenvironment [18-20] and is sustained by the release of factors, such as transforming growth factor-β (TGF-β), bone morphogenetic proteins (BMPs) and bone sialoprotein (BSP), due to tumor-induced activity of osteoclasts [11,21,22]. Satcher and colleagues suggested a crucial role for cadherin-11 in the homing/retention of RCC cells to bone and their subsequent proliferation. By using an *in vivo* metastasis model of RCC, they revealed that the expression of cadherin-11 was enhanced in in BM-derived 786-O cells, and the knockdown of Cadherin-11 by shRNA reduced cell migration. On the other hand, the expression of several factors, including homing receptor CXCR4, HIF-1α, VEGF, IL-6 and RANKL did not differ between cells metastasizing to bone or other organs [23].

Furthermore, Joeckel *et al*. showed that elevated extracellular calcium levels are associated with increased activity of AKT, PLCγ-1, p38α and JNK, with consequently enhanced migration and proliferation of bone metastasizing RCC cells. This process was blocked by the use of calcium-sensing receptor (CaSR) inhibitor NPS 2143, thus suggesting a predictive role of CaSR and its pathway for RCC bone metastases [24].

Recent evidences suggest that BMs have a negative impact on clinical outcomes in patients with mRCC, even worse than the prognosis of patients with liver metastases [8,9]. Some authors suggest that bone metastases may have also a predictive significance, particularly with anti-VEGF-targeted therapy [18,25-27], but this observation requires further investigations and a formal validation. In addition, the role of bisphosphonates in association with targeted therapy in these patients is still controversial [26,28,29].

Santini *et al.* screened the records of more than 1800 patients who died from RCC, finding 398 patients (22%) with BMs [7]. They showed that the majority of patients with BMs at the time of RCC diagnosis were classified as poor risk according to MSKCC criteria, while most of good and intermediate risk patients developed BMs after, respectively, 24 and 5 months.

Based on these findings, can we consider the presence BMs always associated with poor prognosis in RCC patients? Our study aimed to identify existing prognostic factors affecting the outcome of patients with BMs. We showed that age ≥ 65 years, ECOG-PS < 2 and clear cell histology were associated with longer survival. Moreover, MSKCC risk group and ECOG-PS were independent prognostic factors at multivariate analysis. These data are even more interesting if we consider that elderly RCC patients as well as patients with ECOG-PS > 2 or non-clear cell histology have been commonly excluded from clinical trials on the use of targeted agents or bisphosphonates, thus hampering the development of effective therapies for these patients.

We also reported the incidence and prognostic role of concomitant metastases in patients with BMs. We showed that lung, lymph node and liver were the most common sites of concomitant metastases. The presence of lymph-node and/or lung metastases were independent prognostic factors in patients with BMs. Interestingly, the presence of concomitant liver metastases was not an independent prognostic factor in our population, suggesting that future attempts are required in order to optimize the management of patients with concomitant liver and BMs.

We also found that concomitant lung metastases were more frequent in patients with TTBM < 1, and patients with TTBM >5 years had significant longer survival than patients with TTBM < 1 or between 1 and 5 years.

The main limitations of this study include its retrospective design, which is susceptible to bias in data selection and analysis and the heterogeneity of standardized methods used to detect BMs, with each methodology having its own limit of detection.

Despite these limitations, our study suggests that age, ECOG-PS, MSKCC score, TTBM and the presence of concomitant metastases should be necessary considered in order to optimize the prognosis of patients with RCC and BMs. The understanding of the molecular phenotypes of BM-initiating cells in RCC could play a fundamental role in developing therapeutic strategies for these patients.
